# Measles

**Published:** 1884-02

**Authors:** 


### MEASLES.

Prof. Henock (*Ber. Klin. Wochenschrift, Phys. and Surg.*) reports the following: A four-year-old girl was sick with the measles. The eruption came on normally; but the fever did not abate. On the third day after the appearance of the eruption, the entire surface of the body was covered with blisters from the size of a hazel-nut to that of a dollar. The measles exanthem, which could be seen between these bullæ, were hemorrhagic. The whole face was greatly bloated, and the eyelids were so swollen that the eyes could not be opened. After the vesicular eruption ceased, the morning temperature fell to 37.8° C., but the evening temperature rose to 38.5°. The child was threatened with collapse, but revived. However, between the sixth and seventh days, croupous pneumonia set in, and, after eight days, terminated fatally. Similar complications have been reported by Klupfel and Steiner. Henock pronounces this a complication of measles with acute pemphigus.—*Weekly Med. Review.*

